# Genomic Epidemiology and Heterogeneity of SRLV in Italy from 1998 to 2019

**DOI:** 10.3390/v13122338

**Published:** 2021-11-23

**Authors:** Moira Bazzucchi, Ilaria Pierini, Paola Gobbi, Silvia Pirani, Claudia Torresi, Carmen Iscaro, Francesco Feliziani, Monica Giammarioli

**Affiliations:** 1 Istituto Zooprofilattico Sperimentale Umbrita-Marche “Togo Rosati”, 06126 Perugia, Italy; m.bazzucchi@izsum.it (M.B.); i.pierini@izsum.it (I.P.); p.gobbi@izsum.it (P.G.); s.pirani@izsum.it (S.P.); c.torresi@izsum.it (C.T.); c.iscaro@izsum.it (C.I.); f.feliziani@izsum.it (F.F.); 2 Istituto Zooprofilattico Sperimentale della Lombardia e dell’Emilia Romagna “Bruno Ubertini”, 27100 Pavia, Italy

**Keywords:** SRLV, genomic heterogeneity, phylogenetic analyzes

## Abstract

Small ruminant lentiviruses (SRLV) are viruses that retro-transcribe RNA to DNA and show high rates of genetic variability. SRLV affect animals with strains specific for each host species (sheep or goats), resulting in a series of clinical manifestations depending on the virulence of the strain, the host’s genetic background and farm production system. The aim of this work was to present an up-to-date overview of the genomic epidemiology and genetic diversity of SRLV in Italy over time (1998–2019). In this study, we investigated 219 SRLV samples collected from 17 different Italian regions in 178 geographically distinct herds by CEREL. Our genetic study was based on partial sequencing of the *gag-pol* gene (800 bp) and phylogenetic analysis. We identified new subtypes with high heterogeneity, new clusters and recombinant forms. The genetic diversity of Italian SRLV strains may have diagnostic and immunological implications that affect the performance of diagnostic tools. Therefore, it is extremely important to increase the control of genomic variants to improve the control measures.

## 1. Introduction

Small ruminant lentiviruses (SRLV) are heterogenic retroviruses belonging to the *Lentivirus* genus of the *Retroviridae* family [1].

SRLV include two related retroviruses, Visna-maedi virus (VMV) and caprine arthritis and encephalitis virus (CAEV), which were considered separately until a few years ago, while today they represent the prototypes of the two predominant genotypes (A and B1). SRLV are capable of infecting both sheep and goats, especially in mixed flocks; however, some subtypes show a certain specific target adaptation, although they are not considered strictly host-associated [2,3,4].

SRLV induce a multi-systemic disease with progressive inflammatory lesions in the mammary gland, lungs, joints and brain. In one-third of infected animals, symptoms such as pneumonia, arthritis and mastitis have been commonly observed [5].

These single-stranded RNA viruses are responsible for a persistent and lifelong infection by targeting the monocytes of the host and stem cells located in the bone marrow [2].

Genetic variability is a key feature of the SRLV genome, and its knowledge, in addition to shedding light on host-virus interactions, is essential for accurate diagnosis and molecular epidemiology studies. SRLV *quasi-species* are continuously generated through mutation, recombination and selection pressure by the host immune system [6].

Small ruminant variability is a tool for the virus to evade the host immune response and ensure the persistence of the infection [7,8], and may be involved in the crossing of the species barrier. The high rate of variability is revealed by the presence of a number of viral subtypes with variable pathogenic properties within each species [9].

Based on the classification proposed by Shah et al. [10], SRLV have been classified into five genetic groups (A–E), which differ from each other in 25–37% of their nucleotide sequences. Genotypes A, B and E, originally described in sheep or goats, may be distributed into different subtypes (A1–A22, B1–B5 and E1–E2). New subtypes constantly appear as more local strains are analyzed, which outlines the continuous need for surveillance of diagnostic and vaccination strategies [11,12].

New partial sequences have been described in America, Europe and Asia [13,14,15,16]. Genotypes B, C and D and some A subtypes (A1, A3, A4, A5, A6, A9, A11, A12 and A13) have been found in both sheep and goats, while other subtypes have only been reported in sheep (A2, A15, A16) or goats (A7, A8, A10, A14, A17, E1 and E2) [6,13,14]. Recently, new subtypes (A18 and A19) have been found in sheep and goats [4,15], while the subtypes A21/A22 have been found in German and Iranian sheep [13].

In Italy, previous studies on the genetic diversity of SRLV provided evidence for the presence of at least three different genotypes (A, B and E) and 12 subtypes within SRLV [16,17,18,19,20]. According to the taxonomic classification proposed by Shah et al. [10], most of the Italian samples described in sheep and goats clustered into A4, A6, A8, A9, A11, A19, A20, B1, B2, B3, E1 and E2 [13,16,17,18,19,20]. However, the circulation of different variants of SRLV have been reported in Italian territory, also thanks to the recent development of new NGS sequencing technologies [4].

The aim of this work was to report an up-to-date overview of the genomic epidemiology and genetic diversity of SRLV in an Italian small ruminant population over time (1998–2019) by targeting a conserved genome region.

## 2. Materials and Methods

**Figure 1:**
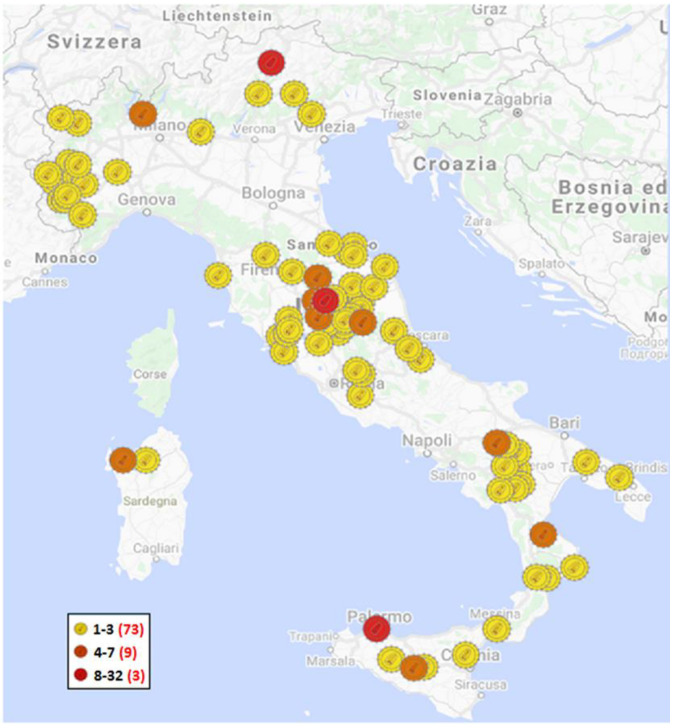
Geographical distribution of SRLV samples analyzed in this study on a municipality basis; the yellow markers show the municipalities in which up to 3 isolates have been identified; the orange markers show the municipalities in which 4 to 7 isolates have been identified; the red markers show the municipalities in which more than 8 isolates have been identified. In the legend, a red number in brackets indicates how many municipalities are included in each category.

The 219 sequences analyzed in this study were collected from 17 Italian regions (178 different flocks): Abruzzo (6); Basilicata (18); Calabria (9); Campania (1); Emilia-Romagna (2); Lazio (5); Lombardia (5); Marche (13); Piemonte (14); Puglia (2); Sardegna (8); Sicilia (15); Toscana (17); Trentino-Alto Adige (32); Umbria (69); Veneto (2); and Valle d’ Aosta (1) over a 22-year period of 1998–2019 (Supplementary Table S1) by the National Reference Centre for Retrovirus (CEREL). These sequences (54) were obtained from a previous study [17] and from viruses (165) sampled from naturally infected sheep and goats and sent to the laboratory for diagnostic purposes. These animals came from 178 geographically distinct herds of sheep, goats or mixed flocks (Figure 1). Other 50 SRLV Italian sequences were considered in the analysis (Supplementary Table S1). Our genetic study was based on the partial *gag-pol* gene fragment (800 bp), as previously described by Shah C. et al., 2004 [10]. We analyzed an 800-bp partial *gag-pol* gene fragment sequenced from proviral SRLV DNA.

Viral RNA or proviral DNA was extracted from the original biological samples (buffy coat, milk or tissues) and identified as SRLV-positive using the QIAamp^®^ RNaesy Mini kit or QIAamp^®^ DNA Mini kit (Qiagen, Valencia, CA, USA) following the manufacturer’s recommendations.

The 800 bp fragment was amplified using a nested-PCR protocol described by Grego et al. [18]. After gel electrophoresis, PCR products from each sample were purified using the QIAquick Gel Extraction kit (Qiagen, Valencia, CA, USA) and used as templates for sequencing with the Big Dye Terminator v3.1 Cycle Sequencing kit (Applied Biosystems, Foster City, CA, USA). Precipitated products were sequenced on an ABI PRISM 3130 Genetic Analyzer (Applied Biosystems, Foster City, CA, USA). Both sense and antisense strands were sequenced by performing three independent reactions for each sample.

The sequence dataset was analyzed using the DNAStar package v.15. Nucleotide sequences were aligned using the Clustal X two algorithm with respect to the amino acid coding frame with 50 published SRLV reference strains retrieved from PubMed at the National Center for Biotechnology Information (NCBI) (http://www.ncbi.nlm.nih.gov/, accessed on 5 October 2021).

Manual editing was performed using BioEdit software (version 7.0.) [21]. The phylogeny was estimated using maximum likelihood (ML) analysis and Bayesian inference (BI) analysis to improve the robustness of the analysis. Maximum analysis (ML) analysis was performed in MEGA v.X [22] using the GTR statistical model with gamma distribution + I (G+I). The robustness of the clusters was assessed by performing 10,000 bootstrap replicates, and branches with bootstrap values exceeding 70% were grouped together. BI analysis was evaluated in BEAST v.1.8.4 with the GTR+G+I substitution model, with two runs consisting of four Markov chains [23]. A consensus tree was constructed using TreeAnnotator v.1.8.4, and the trees were displayed and edited using FigTree v.1.4.0.

The within-group mean distance, between-group mean distance and pairwise distance were calculated in MEGA v.X [22], using the p-distance method (G+I) with 1000 bootstrap replicates.

To detect the occurrence of recombination, a dataset including 319 sequences of SRLV was analyzed using SplitsTree4 [24] and RDP3 [25] software. Recombination events were assessed using the Phi test of SplitsTree v. 4.

## 3. Results

**Figure 2:**
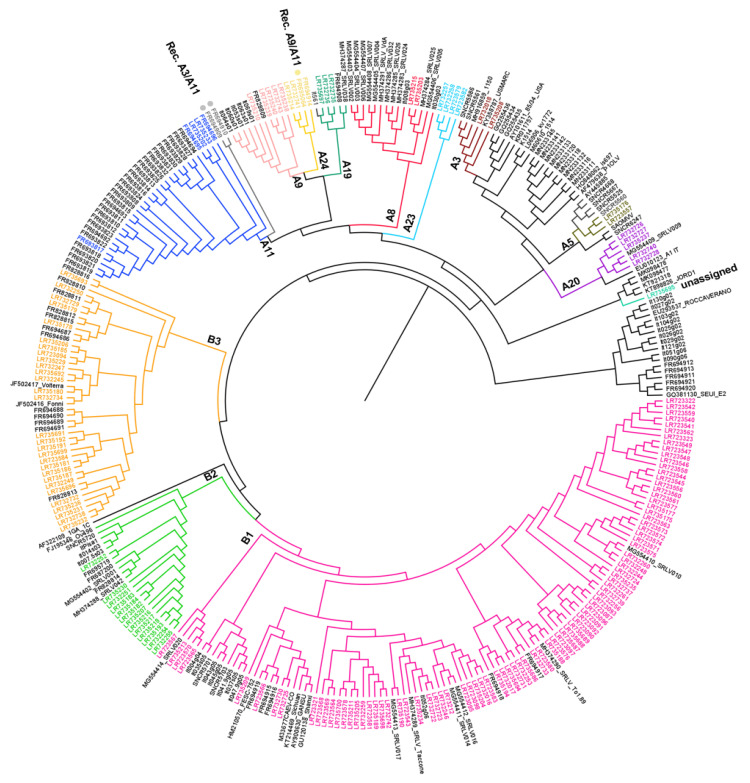
Phylogenetic tree constructed using 640 nt from the SRLV *gag-pol* region of 319 Italian samples and reference strains retrieved from the GenBank database. Bar: number of substitutions per site. Newly characterized samples are reported in colors. Recombinant samples are market with a dot (•).

The topology of the tree obtained (Figure 2) indicates that the 269 Italian samples analyzed in this study belonged to 10 previously described subtypes, two subtypes not previously described in Italy and samples with very high heterogeneity (unassigned). We also described three samples that were characterized by recombination events. This data provides evidence for natural recombination events.

In particular, the clusters already described are A8 (n = 15), A9 (n = 12), A11 (n = 32), A19 (n = 5), A20 (n = 6), B1 (n = 106), B2 (n = 19), B3 (n = 43), E1 (n = 11) and E2 (n = 5) (Supplementary Table S1, Figure 2). Two subtypes, A3 and A5, have not been described in Italy so far (Figure 2). Subtype A3 included two samples (159_BA_2014/LR732018 and 297_LZ_2018/LR735208), which were collected from sheep from two different regions. These samples displayed an intragroup mean distance of 11.20% and the new group members showed an identity percentage range of 87.3–89.0% and 86.5–88.5%, respectively. Similarly, subtype A5 included two samples (113_TR_2012/LR723557 and 255_TR_2018/LR735176) identified in goats in the same geographic region but were epidemiologically unrelated. These samples displayed a within-group mean distance of 0.079 and the new group members showed an identity percentage range of 89.6–90.3% and 89.6–95.8%, respectively. A3 and A5 showed a between-group mean distance range of 0.0126–0.315 and 0.149–0.296, respectively.

Interestingly, four samples (134_SI_2012/LR723582, 224_CA_2016/LR732258, 223_CA_2016/LR732257 and 111_SI_2012/LR723579) differed significantly from all the SRLV clusters described previously (Figure 2). The samples have been detected in sheep and goats, respectively, in different geographical areas and time, and clustered together into a new putative phylogenetic group, tentatively named ‘A23′, based on the tree topology and p-distance values. The new subtype A23 showed a mean distance range of 0.177–0.310 in respect to the other subtype from group A. In particular, the sample 111_SI_2012/LR723579 showed high heterogeneity with other samples from the same cluster (0.147–0.170).

Other four samples, 250_UM_2017/LR732741, 307_LZ_2018/LR735214, 10298/MA/09/FR695064 and 10298/MA/09/FR694914, seem to make a well-defined new subtype based on the topology of the tree and the p-distance values (0.152–0.313); therefore, we tentatively named it subtype ‘A24’.

Based on the results available, the sample FR694914 has been previously classified as A9 [18], but now it clearly belongs to subtype A24.

In addition, a single sample (160_CA_2014/LR735695) collected from a sheep bulk milk from Calabria in 2015 belonged to genotype A, and is not clustered in any of the already known subtypes, showing a between subtypes/groups mean distance value range of 0.189–0.330.

Eighty-seven samples, collected from goats and only one in sheep, clustered in B1 with a within-subtype mean distance of 0.128. This subtype is the most widespread from a geographical point of view based on available data, as it was identified in 17 regions: Abruzzo (3), Basilicata (3), Calabria (1), Emilia Romagna (2), Lombardia (5), Marche (3), Piemonte (9), Sardegna (1), Puglia (1), Sicilia (13), Toscana (2), Trentino (30), Umbria (11), Veneto (2) and Valle d’Aosta (1). Thirteen samples, collected only from sheep in six different regions, clustered in B2, showing a within-group mean distance of 0.082. Twenty-nine samples clustered in subtype B3 showed a higher variability than the other samples of genotype B, showing a within-group mean distance of 0.147.

The genotype E has not been detected within the 165 SRLV samples sequenced in the study, but it has been identified in a preliminary study [17] and in others that described SRLV [18].

**Figure 3:**
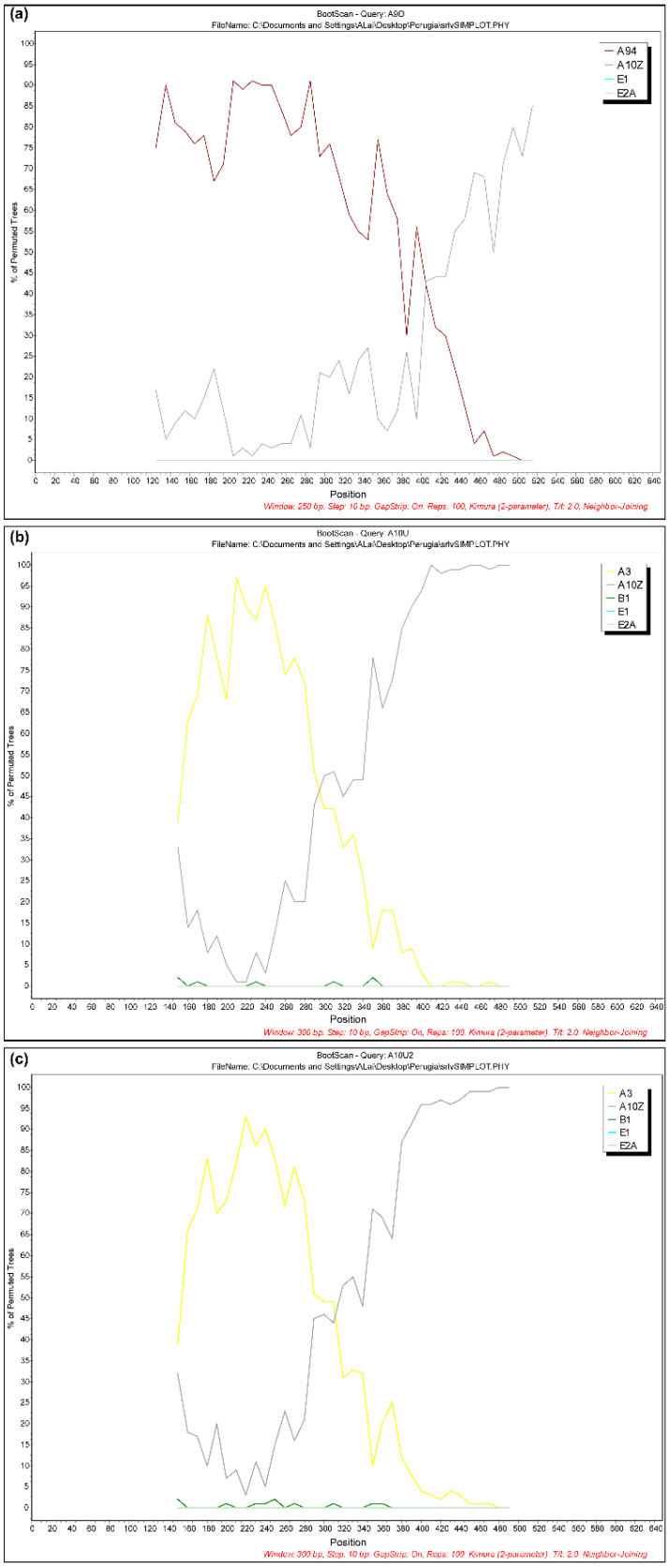
Bootscanning analysis of three samples: (**a**) 51937/UM/06 showed an A9/A11 pattern; (**b**) 10308/MA/09 and (**c**) 10310/MA/09 showed a similar A3/A9 mosaic structure. Non-recombinant reference genomes from subtypes A through E were included in the analysis. Analyses were performed using a window size of 250 or 300 nt and a step size of 10 nt. The *x*-axis indicates nucleotide positions along the alignment. The *y*-axis indicates the percentage of bootstrap values between the query and the background references.

The presence of recombinant strains was investigated using RDP3, Simplot and Splits Tree programs. Interestingly, the results showed evidence of natural recombinant signals in three virus samples collected from different geographical areas, animal species and time. In particular, one virus sample (51937/UM/06/FR695064) showed an A9/A11 pattern, while two samples (10308/MA/09/FR694909 and 10310/MA/09/FR694910) had a similar A3/A10 mosaic structure (Figure 3).

## 4. Discussion

SRLV infection has a considerable economic effect on sheep and goat breeding; nevertheless, its impact on small ruminant production is largely underestimated by local farmers. Currently, there are no treatments or vaccines against SRLV. Live trade of goats or sheep from different countries or regions where the disease has been reported is believed to be the main reason for its wide spread. Thus, control programs remain the only way to avoid the spread of the SRLV infection [6]. In most countries there is no particular attention to SRLV infections. Up to now, there have been sporadic control plans mostly limited to goats and the B genotype circulation in areas characterized by particular food productions. In Europe, control programs have been implemented in many countries since SRLV have been detected in their goatherds [26,27,28,29].

In Italy, there is no national mandatory SRLV control and eradication plan; however, some regions, due to the large number of small ruminant farms—and therefore, being concerned about the problem —have autonomously devised initiatives to receive a SRLV-free status [30]. There are few voluntary and sporadic mandatory eradication programs on a local basis aimed at the eradication of CAEV [31,32,33].

The accurate diagnosis of the SRLV infection is of major importance in epidemiological research, control programs and safe small ruminant international trade according to the World Organization for Animal Health (OIE) recommendations. These objectives might be hampered by the high genetic and biological variation of SRLV; in general, however, such infections are efficiently detected through serological methods that can be complemented with molecular techniques [34].

Small ruminant lentivirus *quasi-species* are continuously generated through mutation, recombination and selection pressure by the host immune system. New subtypes constantly appear as more local strains are analyzed, which outlines the continuous need for surveillance of diagnostic designs [11,12].

In this study, we have set the goal of outlining an image as comprehensively as possible of the situation in Italy regarding SRLV, expanding the study in terms of the territories involved and the time of observation.

The current SRLV phylogeny, consisting of five genotypes, which are further divided into multiple subtypes, emphasizes the high genetic variation among SRLV strains.

Although there are other molecular studies based on different genomic virus regions [10,35,36], the scientific approach based on sequencing of the *gag-pol* region (800 bp) is the one most used by different scientific groups [10,16,17,18,20]. Based on this approach, SRLV were classified in the following genotypes/sub types: A1, A2, A3, A4, A5, A8, A9, A11, A19, A20, A21, A22, C, B1, B2, B3, E1 and E2. For completeness, the SRLV genotypes/subtypes were compared with samples that are present in the GenBank database using a shorter overlapping *gag* region (420 bp). These analyses are less informative, as with the shorter alignment we lost recombination information; nevertheless, we confirmed that none of the Italian samples considered in this study clustered within other described subtypes (A12, A13, A16, A17 and A18; data not shown).

Genotype B contains three subtypes: B1, B2 and B3. The B4 subtype described by Santry et al., 2013 [37] was previously classified as a recombinant group within genotype A [38]. Subtype B5 was classified based on the sequences of the *pol* region, but it was classified as B1 based on the overlapping *gag* region [39].

Group C strains, originally sampled from Norwegian goats and group E strains are characterized by their extensive genetic divergence from other SRLV groups and their limited geographical range [10,18,40]. Genotype D was found in a few samples originating from Switzerland and Spain, but is now reclassified as genotype A [13]. Group E strains are characterized by their extensive genetic divergence from other SRLV groups and their limited geographical range [10,18,40].

This high genetic diversity between strains often poses challenges for countries that implement SRLV eradication programs, as none of the existing diagnostic tests are capable of detecting all circulating strains [3].

The sequences obtained from this study were analyzed with all the SRLV genotypes previously described, but only the results obtained with the *gag-pol* region (800 bp) are shown in Figure 2. The phylogenetic tree based on the *gag* (420 nt) region is not shown.

As previously reported, we identified seven described A subtypes (A3, A5, A8, A9, A11, A19 and A20). The subtypes A12, A13, A16, A17 and A18 were not identified in this study.

Two subtypes, A3 and A5, previously reported in Spain, Turkey, Switzerland and Germany, Slovenia and Poland [13,36,41] have been identified as sporadic events in Italy based on data analyzed. Seven sequences are placed in two distinct putative new clusters, (134_SI_2012/LR723582, 224_CA_2016/LR732258, 223_CA_2016/LR732257, 111_SI_2012/LR723579 and 250_UM_2017/LR732741, 307_LZ_2018/LR735214, 10298/MA/09/FR695064), named ‘A23′ and ‘A24′ respectively.

The subtypes A23 and A24 are quite distant from each other; in particular, the most distant from the other A subtypes in the branch was A23. It includes four sequences (three from sheep, one from goat) retrieved from two neighboring regions that are very similar regarding the types of farms present (Sicilia and Calabria). The A24 includes three sequences obtained from three neighboring regions of central Italy (Umbria, Marche and Lazio), between which there are frequent commercial exchanges. These regions are epidemiologically related because of common trade practices between sheep and goat farmers.

The sequence 160_CA_2014/LR735695 has been identified in bulk milk collected from a mixed outdoor farm (Calabria), characterized by mixed breeding and crossroads of different races. The sample showed high diversity compared to the other genotype A samples, but a high homology with the cluster identified by Molaee et al. [13] and defined as Middle Eastern/Iranian. This cluster was characterized through a high nucleotide identity with the samples considered to be ancestors of SRLV.

Finally, three samples (51937/UM/06/FR695064, 10308/MA/09/FR694909 and 10310/MA/09/FR694910) showed natural recombination events.

The genetic diversity of lentiviruses is driven by a low-fidelity reverse transcriptase and their propensity to recombine via strand transfer [42]. Co-circulation and co-infection with more than one lentivirus type offers an opportunity for viral recombination. Only a handful of reports of mixed infections and recombination under both experimental and natural conditions have been published to date [34,43,44,45,46,47,48,49]. Further studies are necessary to understand the role of natural recombinants in the spread of SRLV, as well as their ability to evade the current diagnostic tests.

Most of the Italian samples characterized in this study (130) belonged to the most homologous genotype B. Subtype B1 is the most represented in general and this study contributes to this evidence. In fact, 88 samples clustered in subtype B1. Although subtype B1 has been identified in six sheep, it represents the main genotype for goats.

B2 and B3 are subtypes that mainly affect sheep: 19 out of 94 sequences of ovine origin showed B2 subtype and 38 were subtype B3. The latter viral subtype was also detected in five samples of goat origin.

It is important to observe the great variability within B1 and B3, in which well-defined internal clusters branch out. Although the calculated mean distance in the groups appears to be quite high, the BI does not support further separation. For genotype E, the results agreed with those already published in our preliminary molecular epidemiological study. This genotype is not widespread and describes a type of SRLV that is non-pathogenic or has low pathogenicity.

The great SRLV heterogeneity in Italy can also be explained by the presence of different herd management, different breeding systems, transhumance, pastoring practices, micro environmental factors and habitat. The geographical distribution, presence of wild ungulates and climate change may also be involved.

In summary, this work provides new knowledge on the circulation of SRLV variants circulation in Italy over a long period, and provides new information about the presence of never-identified subtypes.

The rapid evolution of SRLV and emergence of new recombinant forms, subtypes and groups may have significant implications for the development of reliable diagnostic testing and in the success of eradication programs. To better understand the impact of the high divergence detected, it might be useful to extend the analysis to the full genome, at least for some selected samples. The genetic heterogeneity amongst field strains of SRLV remains to be fully characterized, which is important as this genetic variation in turn translates into virus strains and genetic sequences with different biological properties such as virulence.

The current situation of eradication and control programs for SRLV in the ovine/caprine population is underestimated in Italy. For SRLV mitigation, it is extremely important to regulate the animal trade according to the disease status of a farm or region, and to increase the control and restriction of trade of biological products.

The genetic characterization of Italian SRLV strains will help in the development of appropriate diagnostic tools to assist in the national control program. Interestingly, the study shows the need for a new classification, not necessarily based on complete sequences, but taking into account all the cases of a dubious clustering based on the current classification.

## Data Availability

Accession numbers: The partial SRLV sequences generated in this study have been deposited in the NCBI GenBank database www.ncbi.nlm.nih.gov (Supplementary Table S1).

## Supplementary Materials

The following are available online at https://www.mdpi.com/article/10.3390/v13122338/s1, Table S1: Dataset description of SRLV samples analysed in the study.
