# Supplementary material for: Genomic Epidemiology and Heterogeneity of SRLV in Italy from 1998 to 2019
Source: Viruses. 2021 Nov 23;13(12):2338. doi: 10.3390/v13122338 (PMC8706641; doi:10.3390/v13122338)
Supplement: Supplementary file 1 [file viruses-13-02338-s001.zip › viruses-1448782-supplementary.pdf]

**Table S1: Dataset description of SRLV samples analysed in the study.**

| ID strain | Year | Region | Species | Accession number | Genotypes /subtypes | Reference |
|---|---|---|---|---|---|---|
| SRLV_To1.89 | 1989 | Piemonte | Goat | MH374290 | B1 | Bertolotti L., et al 2018 |
| 1G/SA/98 | 1998 | Sardegna | Goat | FR694915 | B1 | Giammarioli M. et al 2011 |
| TC2/SA/99 | 1999 | Sardegna | Goat | FR694916 | B1 | Giammarioli M. et al 2011 |
| 414/UM/00 | 2000 | Umbria | Sheep | FR694696 | A11 | In this study |
| 22803.29.UM.10 | 2000 | Umbria | Goat | FR693817 | A11 | In this study |
| It561 | 2005 | Piemonte | Sheep | AY265455 | A19 | Grego E et al 2005 |
| 7008/UM/01 | 2001 | Umbria | Sheep | FR695719 | B2 | Giammarioli M. et al 2011 |
| 74528.6/SA/02 | 2002 | Sardegna | Goat | FR694920 | E2 | Giammarioli M. et al 2011 |
| 74528.13/SA/02 | 2002 | Sardegna | Goat | FR694921 | E2 | Giammarioli M. et al 2011 |
| Fonni | 2003 | Sardegna | Sheep | JF502416 | B3 | Bertolotti L., et al 2011 |
| Volterra | 2004 | Toscana | Sheep | JF502417 | B3 | Bertolotti L., et al 2011 |
| It-008g03 | 2006 | Piemonte | Goat | EU010125 | A8 | Grego E., et al 2007 |
| It-014s03 | 2006 | Piemonte | Sheep | EF676000 | B2 | Grego E., et al 2007 |
| It-025g02 | 2006 | Piemonte | Goat | EU010124 | E1 | Grego E., et al 2007 |
| It-026g02 | 2006 | Piemonte | Goat | EF676003 | E1 | Grego E., et al 2007 |
| It-027g02 | 2006 | Piemonte | Goat | EF676004 | E1 | Grego E., et al 2007 |
| It-029g02 | 2006 | Piemonte | Goat | EF676005 | E1 | Grego E., et al 2007 |
| It-030g03 | 2006 | Piemonte | Goat | EF676006 | A8 | Grego E., et al 2007 |
| It-035s05 | 2006 | Piemonte | Sheep | EF676007 | B1 | Grego E., et al 2007 |
| It-037s05 | 2006 | Piemonte | Sheep | EF676009 | B1 | Grego E., et al 2007 |
| It-043g05 | 2006 | Piemonte | Goat | EU010121 | B1 | Grego E., et al 2007 |
| It-045g05 | 2006 | Piemonte | Goat | EF676013 | B1 | Grego E., et al 2007 |
| It-047g05 | 2006 | Piemonte | Goat | EU010120 | B1 | Grego E., et al 2007 |
| It-051g06 | 2006 | Piemonte | Goat | EF676014 | E1 | Grego E., et al 2007 |
| It-053s01 | 2006 | Piemonte | Sheep | EF676016 | A9 | Grego E., et al 2007 |
| It-052g06 | 2006 | Piemonte | Goat | EF676015 | B1 | Grego E., et al 2007 |
| It-090g06 | 2006 | Piemonte | Goat | EF676021 | E1 | Grego E., et al 2007 |
| It-069s01 | 2006 | Piemonte | Sheep | EF676020 | A9 | Grego E., et al 2007 |

| | | | | | | |
|---|---|---|---|---|---|---|
| It-064s01 | 2006 | Piemonte | Sheep | EF676019 | A9 | Grego E., et al 2007 |
| It-063s01 | 2006 | Piemonte | Sheep | EF676018 | A9 | Grego E., et al 2007 |
| It-060s01 | 2006 | Piemonte | Sheep | EF676017 | A9 | Grego E., et al 2007 |
| 32214/UM/06 | 2006 | Umbria | Sheep | FR694691 | B3 | Giammarioli M. et al 2011 |
| 51937/UM/06 | 2006 | Umbria | Goat | FR695064 | recombinant A9/A11 | In this study |
| It-103g02 | 2007 | Piemonte | Goat | EF676022 | E1 | Grego E., et al 2007 |
| It-130g02 | 2007 | Piemonte | Goat | EF676025 | E1 | Grego E., et al 2007 |
| It-121g02 | 2007 | Piemonte | Goat | EF676024 | E1 | Grego E., et al 2007 |
| It-104g02 | 2007 | Piemonte | Goat | EF676023 | E1 | Grego E., et al 2007 |
| 04/TO/08 | 2008 | Toscana | Goat | FR694917 | B1 | Giammarioli M. et al 2011 |
| 380/TO/08 | 2008 | Toscana | Goat | FR694918 | B1 | Giammarioli M. et al 2011 |
| Roccaverano | 2008 | Piemonte | Goat | EU293537 | E1 | Reina R. et al 2009 |
| 10294/MA/09 | 2009 | Marche | Goat | FR694689 | B3 | Giammarioli M. et al 2011 |
| 10298/MA/09 | 2009 | Marche | Sheep | FR694914 | A24 | In this study |
| 10308/MA/09 | 2009 | Marche | Sheep | FR694909 | recombinant A3/A11 | In this study |
| 63088/UM/09 | 2009 | Umbria | Goat | FR694919 | B1 | Giammarioli M. et al 2011 |
| ME.24/UM/09 | 2009 | Umbria | Goat | FR694912 | E2 | Giammarioli M. et al 2011 |
| 6770/UM/09 | 2009 | Umbria | Goat | FR693815 | A11 | Giammarioli M. et al 2011 |
| 5480.2/SA/09 | 2009 | Sardegna | Sheep | FR694686 | B3 | Giammarioli M. et al 2011 |
| 5480.10/SA/09 | 2009 | Sardegna | Sheep | FR694687 | B3 | Giammarioli M. et al 2011 |
| 36384.1/SA/09 | 2009 | Sardegna | Goat | FR694688 | B3 | Giammarioli M. et al 2011 |
| 06/PI/09 | 2009 | Piemonte | Sheep | FR687200 | B2 | Giammarioli M. et al 2011 |
| 10310/MA/09 | 2009 | Marche | Sheep | FR694910 | recombinant A3/A11 | In this study |
| 2803.35/UM/10 | 2010 | Toscana | Goat | FR693818 | A11 | Giammarioli M. et al 2011 |
| 5012/UM/10 | 2010 | Umbria | Goat | FR693819 | A11 | Giammarioli M. et al 2011 |
| 6440/UM/10 | 2010 | Umbria | Goat | FR693820 | A11 | Giammarioli M. et al 2011 |
| 1810/UM/10 | 2010 | Umbria | Goat | FR693821 | A11 | Giammarioli M. et al 2011 |
| 2803.34/UM/10 | 2010 | Toscana | Goat | FR693822 | A11 | Giammarioli M. et al 2011 |
| 2797.4/UM/10 | 2010 | Toscana | Sheep | FR694692 | A11 | Giammarioli M. et al 2011 |

| | | | | | | |
|---|---|---|---|---|---|---|
| 2607/UM/10 | 2010 | Umbria | Goat | FR693823 | A11 | Giammarioli M. et al 2011 |
| 0222/UM/10 | 2010 | Umbria | Goat | FR693810 | A11 | Giammarioli M. et al 2011 |
| 2798.4/UM/10 | 2010 | Umbria | Goat | FR693812 | A11 | Giammarioli M. et al 2011 |
| 2803.32/UM/10 | 2010 | Toscana | Goat | FR693811 | A11 | Giammarioli M. et al 2011 |
| 2803.42/UM/10 | 2010 | Toscana | Goat | FR693809 | A11 | Giammarioli M. et al 2011 |
| 2803.28/UM/10 | 2010 | Toscana | Goat | FR693808 | A11 | Giammarioli M. et al 2011 |
| 2356/UM/10 | 2010 | Umbria | Goat | FR693824 | A11 | Giammarioli M. et al 2011 |
| 2797.11/UM/10 | 2010 | Toscana | Sheep | FR694693 | A11 | Giammarioli M. et al 2011 |
| 6780/UM/10 | 2010 | Umbria | Goat | FR693816 | A11 | Giammarioli M. et al 2011 |
| 1462/UM/10 | 2010 | Umbria | Goat | FR693814 | A11 | Giammarioli M. et al 2011 |
| 2393/UM/10 | 2010 | Umbria | Goat | FR693813 | A11 | Giammarioli M. et al 2011 |
| 7995/UM/10 | 2010 | Umbria | Goat | FR693832 | A11 | Giammarioli M. et al 2011 |
| 5065/UM/10 | 2010 | Umbria | Goat | FR693831 | A11 | Giammarioli M. et al 2011 |
| 7978/UM/10 | 2010 | Umbria | Goat | FR693830 | A11 | Giammarioli M. et al 2011 |
| 6408/UM/10 | 2010 | Umbria | Goat | FR693829 | A11 | Giammarioli M. et al 2011 |
| 2357/UM10 | 2010 | Umbria | Goat | FR693827 | A11 | Giammarioli M. et al 2011 |
| 4150/UM/10 | 2010 | Umbria | Goat | FR693828 | A11 | Giammarioli M. et al 2011 |
| 2797.1/UM/10 | 2010 | Toscana | Sheep | FR694694 | A11 | Giammarioli M. et al 2011 |
| 9440/UM/10 | 2010 | Umbria | Goat | FR693826 | A11 | Giammarioli M. et al 2011 |
| 0245/UM/10 | 2010 | Umbria | Goat | FR693825 | A11 | Giammarioli M. et al 2011 |
| 0249/UM/10 | 2010 | Umbria | Goat | FR694690 | B3 | Giammarioli M. et al 2011 |
| 1017/UM/10 | 2010 | Umbria | Goat | FR694908 | A9 | Giammarioli M. et al 2011 |
| ME.30/UM/10 | 2010 | Umbria | Goat | FR694913 | E2 | Giammarioli M. et al 2011 |
| 2803.25/UM/10 | 2010 | Toscana | Goat | FR694911 | E2 | Giammarioli M. et al 2011 |
| LaSaz06/UM/10 | 2010 | Umbria | Sheep | FR828809 | A9 | Giammarioli M. et al 2011 |
| 11058/UM/10 | 2010 | Umbria | Sheep | FR828810 | B3 | Giammarioli M. et al 2011 |
| 11067/UM/10 | 2010 | Umbria | Sheep | FR828811 | B3 | Giammarioli M. et al 2011 |
| 11063/UM/10 | 2010 | Umbria | Sheep | FR828812 | B3 | Giammarioli M. et al 2011 |

| | | | | | | |
|---|---|---|---|---|---|---|
| 11059/UM/10 | 2010 | Umbria | Sheep | FR828813 | B3 | Giammarioli M. et al 2011 |
| AN13/MA/10 | 2010 | Marche | Sheep | FR828814 | B2 | Giammarioli M. et al 2011 |
| PS075/MA/10 | 2010 | Marche | Sheep | FR828815 | B3 | Giammarioli M. et al 2011 |
| AN10/MA/10 | 2010 | Marche | Sheep | FR828816 | B3 | Giammarioli M. et al 2011 |
| La1SAZ/UM/10 | 2010 | Umbria | Sheep | FR694695 | A11 | In this study |
| 76_SI_2011 | 2011 | Sicilia | Goat | LR723571 | B1 | In this study |
| 77_SI_2011 | 2011 | Sicilia | Goat | LR723578 | B1 | In this study |
| 78_SI_2011. | 2011 | Sicilia | Goat | LR723572 | B1 | In this study |
| 79_SI_2011 | 2011 | Sicilia | Goat | LR723573 | B1 | In this study |
| 80_SI_2011 | 2011 | Sicilia | Goat | LR723574 | B1 | In this study |
| 81_SI_2011 | 2011 | Sicilia | Goat | LR723575 | B1 | In this study |
| 82_SI_2011 | 2011 | Sicilia | Goat | LR723576 | B1 | In this study |
| 83_SI_2011 | 2011 | Sicilia | Goat | LR723087 | B1 | In this study |
| 84_SI_2011 | 2011 | Sicilia | Goat | LR723090 | B1 | In this study |
| 85_SI_2011 | 2011 | Sicilia | Goat | LR723091 | B1 | In this study |
| 86_SI_2011 | 2011 | Sicilia | Goat | LR723092 | B1 | In this study |
| 87_SI_2011 | 2011 | Sicilia | Goat | LR723093 | B1 | In this study |
| 89_LZ_2011 | 2011 | Lazio | Sheep | LR723094 | B3 | In this study |
| 94_UM_2011 | 2011 | Umbria | Goat | LR723095 | B1 | In this study |
| 95_UM_2011 | 2011 | Umbria | Goat | LR723096 | B1 | In this study |
| 96_UM_2011 | 2011 | Umbria | Goat | LR723097 | B1 | In this study |
| 97_UM_2011 | 2011 | Umbria | Goat | LR723098 | B1 | In this study |
| 98_TR_2012 | 2012 | Trentino-AltoAdige | Goat | LR723321 | B1 | In this study |
| 99_TR_2012 | 2012 | Trentino-AltoAdige | Goat | LR723322 | B1 | In this study |
| 100_TR_2012 | 2012 | Trentino-AltoAdige | Goat | LR723323 | B1 | In this study |
| 101_TR_2012 | 2012 | Trentino-AltoAdige | Goat | LR723540 | B1 | In this study |
| 102_TR_2012 | 2012 | Trentino-AltoAdige | Goat | LR723541 | B1 | In this study |
| 103_TR_2012 | 2012 | Trentino-AltoAdige | Goat | LR723542 | B1 | In this study |
| 104_TR_2012 | 2012 | Trentino-AltoAdige | Goat | LR723546 | B1 | In this study |

| | | | | | | |
|---|---|---|---|---|---|---|
| 105_TR_2012 | 2012 | Trentino-AltoAdige | Goat | LR723547 | B1 | In this study |
| 106_TR_2012 | 2012 | Trentino-AltoAdige | Goat | LR723548 | B1 | In this study |
| 107_TR_2012 | 2012 | Trentino-AltoAdige | Goat | LR723549 | B1 | In this study |
| 108_TR_2012 | 2012 | Trentino-AltoAdige | Goat | LR723545 | B1 | In this study |
| 109_TR_2012 | 2012 | Trentino-AltoAdige | Goat | LR723543 | B1 | In this study |
| 110_TR_2012 | 2012 | Trentino-AltoAdige | Goat | LR723544 | B1 | In this study |
| 111_SI_2012 | 2012 | Sicilia | Goat | LR723579 | A23 | In this study |
| 112_TR_2012 | 2012 | Trentino-AltoAdige | Goat | LR723556 | B1 | In this study |
| 113_TR_2012 | 2012 | Trentino-AltoAdige | Goat | LR723557 | A5 | In this study |
| 114_TR_2012 | 2012 | Trentino-AltoAdige | Goat | LR723558 | B1 | In this study |
| 115_TR_2012 | 2012 | Trentino-AltoAdige | Goat | LR723559 | B1 | In this study |
| 118_TR_2012 | 2012 | Trentino-AltoAdige | Goat | LR723560 | B1 | In this study |
| 119_TR_2012 | 2012 | Trentino-AltoAdige | Goat | LR723561 | B1 | In this study |
| 120_TR_2012 | 2012 | Trentino-AltoAdige | Goat | LR723562 | B1 | In this study |
| 121_TR_2012 | 2012 | Trentino-AltoAdige | Goat | LR723563 | B1 | In this study |
| 122_TR_2012 | 2012 | Trentino-AltoAdige | Goat | LR723564 | B1 | In this study |
| 123_TR_2012 | 2012 | Trentino-AltoAdige | Goat | LR723565 | B1 | In this study |
| 124_TR_2012 | 2012 | Trentino-AltoAdige | Goat | LR723566 | B1 | In this study |
| 125_TR_2012 | 2012 | Trentino-AltoAdige | Goat | LR723567 | B1 | In this study |
| 126_TR_2012 | 2012 | Trentino-AltoAdige | Goat | LR723568 | B1 | In this study |
| 127_TR_2012 | 2012 | Trentino-AltoAdige | Goat | LR723569 | B1 | In this study |
| 128_TR_2012 | 2012 | Trentino-AltoAdige | Goat | LR723570 | B1 | In this study |
| 129_TR_2012 | 2012 | Trentino-AltoAdige | Goat | LR723577 | B1 | In this study |
| 131_SI_2012 | 2012 | Sicilia | Sheep | LR723580 | B1 | In this study |
| 133_VE_2012 | 2012 | Veneto | Goat | LR723581 | B1 | In this study |
| 134_SI_2012 | 2012 | Sicilia | Sheep | LR723582 | A23 | In this study |
| 135_MA_2013 | 2013 | Marche | Goat | LR723583 | B1 | In this study |
| 138_CA_2013 | 2013 | Calabria | Sheep | LR723584 | B3 | In this study |
| 140_MA_2014 | 2014 | Marche | Goat | LR732016 | B1 | In this study |

| | | | | | | |
|---|---|---|---|---|---|---|
| 143_UM_2014 | 2014 | Umbria | Goat | LR732017 | B1 | In this study |
| 144_LO_2014 | 2014 | Lombardia | Goat | LR735686 | B1 | In this study |
| 145_LO_2014 | 2014 | Lombardia | Goat | LR735687 | B1 | In this study |
| 146_LO_2014 | 2014 | Lombardia | Goat | LR735688 | B1 | In this study |
| 147_LO_2014 | 2014 | Lombardia | Goat | LR735689 | B1 | In this study |
| 151_UM_2014 | 2014 | Umbria | Sheep | LR735691 | B3 | In this study |
| 152_UM_2014 | 2014 | Umbria | Sheep | LR735692 | B3 | In this study |
| 155_TO_2014 | 2014 | Toscana | Sheep | LR735693 | B3 | In this study |
| 158_UM_2014 | 2014 | Umbria | Goat | LR735694 | B1 | In this study |
| 159_BA_2014 | 2014 | Basilicata | Sheep | LR732018 | A3 | In this study |
| 160_CA_2014 | 2014 | Calabria | Sheep | LR735695 | Unassigned | In this study |
| 174_UM_2015 | 2015 | Umbria | Sheep | LR735696 | B3 | In this study |
| 179_UM_2015 | 2015 | Umbria | Sheep | LR735697 | A19 | In this study |
| 180_UM_2015 | 2015 | Umbria | Sheep | LR735698 | B1 | In this study |
| 181_BA_2015 | 2015 | Basilicata | Sheep | LR735699 | B3 | In this study |
| 183_PI_2015 | 2015 | Piemonte | Goat | LR735700 | B1 | In this study |
| 185_VE_2016 | 2016 | Veneto | Goat | LR732244 | B1 | In this study |
| 186_BA_2016 | 2016 | Basilicata | Sheep | LR732245 | B3 | In this study |
| 194_VDA_2016 | 2016 | Valle d'Aosta | Goat | LR732246 | B1 | In this study |
| 196_MA_2016 | 2016 | Marche | Sheep | LR732247 | B3 | In this study |
| 199_PU_2016 | 2016 | Puglia | Goat | LR732248 | B1 | In this study |
| 200_LZ_2016 | 2016 | Lazio | Sheep | LR732249 | B3 | In this study |
| 201_LZ_2016 | 2016 | Lazio | Sheep | LR732250 | B3 | In this study |
| 202_TO_2016 | 2016 | Toscana | Sheep | LR732251 | B2 | In this study |
| 208_UM_2016 | 2016 | Umbria | Sheep | LR732252 | B2 | In this study |
| 211_SA_2016 | 2016 | Sardegna | Goat | LR732253 | B1 | In this study |
| 219_AB_2016 | 2016 | Abruzzo | Sheep | LR732254 | B2 | In this study |
| 220_AB_2016 | 2016 | Abruzzo | Sheep | LR732255 | B2 | In this study |
| 221_AB_2016 | 2016 | Abruzzo | Goat | LR732256 | B1 | In this study |
| 223_CA_2016 | 2016 | Calabria | Sheep | LR732257 | A23 | In this study |

| | | | | | | |
|---|---|---|---|---|---|---|
| 224_CA_2016 | 2016 | Calabria | Sheep | LR732258 | A23 | In this study |
| 225_AB_2016 | 2016 | Abruzzo | Goat | LR732259 | B1 | In this study |
| 226_TO_2016 | 2016 | Toscana | Goat | LR732260 | B1 | In this study |
| SRLV001 | 2017 | Piemonte | Sheep | MG554402 | B2 | Colitti B., et al 2017 |
| SRLV002 | 2017 | Piemonte | Goat | MG554403 | A8 | Colitti B., et al 2017 |
| SRLV003 | 2017 | Piemonte | Goat | MG554404 | A8 | Colitti B., et al 2017 |
| SRLV004 | 2017 | Piemonte | Goat | MG554405 | A8 | Colitti B., et al 2017 |
| SRLV005 | 2017 | Piemonte | Goat | MG554406 | A8 | Colitti B., et al 2017 |
| SRLV006 | 2017 | Piemonte | Goat | MG554407 | A8 | Colitti B., et al 2017 |
| SRLV007 | 2017 | Piemonte | Goat | MG554408 | A8 | Colitti B., et al 2017 |
| SRLV009 | 2017 | Piemonte | Goat | MG554409 | A20 | Colitti B., et al 2017 |
| SRLV010 | 2017 | Piemonte | Goat | MG554410 | B1 | Colitti B., et al 2017 |
| SRLV014 | 2017 | Piemonte | Goat | MG554411 | B1 | Colitti B., et al 2017 |
| SRLV016 | 2017 | Piemonte | Goat | MG554412 | B1 | Colitti B., et al 2017 |
| SRLV017 | 2017 | Piemonte | Goat | MG554413 | B1 | Colitti B., et al 2017 |
| SRLV020 | 2017 | Piemonte | Goat | MG554414 | B1 | Colitti B., et al 2017 |
| SRLV024 | 2017 | Piemonte | Goat | MH374283 | A8 | Bertolotti L., et al 2018 |
| SRLV025 | 2017 | Piemonte | Goat | MH374284 | A8 | Bertolotti L., et al 2018 |
| SRLV026 | 2017 | Piemonte | Goat | MH374285 | A8 | Bertolotti L., et al 2018 |
| SRLV032 | 2017 | Piemonte | Goat | MH374286 | A8 | Bertolotti L., et al 2018 |
| SRLV038 | 2017 | Piemonte | Sheep | MH374287 | A19 | Bertolotti L., et al 2018 |
| SRLV042 | 2017 | Piemonte | Sheep | MH374288 | B2 | Bertolotti L., et al 2018 |
| SRLV_Taccone | 2017 | Piemonte | Goat | MH374289 | B1 | Bertolotti L., et al 2018 |
| SRLV_VdA | 2017 | Piemonte | Goat | MH374291 | A8 | Bertolotti L., et al 2018 |
| 227_PI_2016 | 2017 | Piemonte | Goat | LR732261 | B1 | In this study |
| 228_EMR_2017 | 2017 | Emilia Romagna | Goat | LR732722 | B1 | In this study |
| 229_EMR_2017 | 2017 | Emilia Romagna | Goat | LR732723 | B1 | In this study |
| 230_AB_2017 | 2017 | Abruzzo | Goat | LR732724 | B1 | In this study |
| 231_PI_2017 | 2017 | Piemonte | Goat | LR732725 | B1 | In this study |
| 232_UM_2017 | 2017 | Umbria | Goat | LR732726 | A20 | In this study |

| | | | | | | |
|---|---|---|---|---|---|---|
| 235_UM_2017 | 2017 | Umbria | Goat | LR732727 | A20 | In this study |
| 237_PI_2017 | 2017 | Piemonte | Goat | LR732728 | A20 | In this study |
| 238_BA_2017 | 2017 | Basilicata | Sheep | LR732729 | B3 | In this study |
| 239_PI_2017 | 2017 | Piemonte | Sheep | LR732730 | B1 | In this study |
| 240_LO_2017 | 2017 | Lombardia | Goat | LR732731 | B1 | In this study |
| 241_UM_2017 | 2017 | Umbria | Sheep | LR732732 | B3 | In this study |
| 242_PI_2017 | 2017 | Piemonte | Goat | LR732733 | B1 | In this study |
| 243_UM_2017 | 2017 | Umbria | Sheep | LR732734 | B3 | In this study |
| 244_MA_2017 | 2017 | Marche | Goat | LR732735 | A19 | In this study |
| 245_MA_2017 | 2017 | Marche | Goat | LR732736 | A19 | In this study |
| 246_UM_2017 | 2017 | Umbria | Goat | LR732737 | B1 | In this study |
| 247_UM_2017 | 2017 | Umbria | Sheep | LR732738 | B3 | In this study |
| 248_CA_2017 | 2017 | Calabria | Goat | LR732739 | B1 | In this study |
| 249_PI_2017 | 2017 | Piemonte | Goat | LR732740 | A20 | In this study |
| 250_UM_2017 | 2017 | Umbria | Sheep | LR732741 | A24 | In this study |
| 251_PI_2017 | 2017 | Piemonte | Goat | LR732742 | B1 | In this study |
| 253_BA_2017 | 2017 | Basilicata | Sheep | LR732743 | A9 | In this study |
| 254_TR_2018 | 2018 | Trentino-AltoAdige | Goat | LR735175 | B1 | In this study |
| 255_TR_2018 | 2018 | Trentino-AltoAdige | Goat | LR735176 | A5 | In this study |
| 256_TR_2018 | 2018 | Trentino-AltoAdige | Goat | LR735177 | B1 | In this study |
| 257_UM_2018 | 2018 | Umbria | Sheep | LR735178 | B3 | In this study |
| 258_UM_2018 | 2018 | Umbria | Sheep | LR735179 | B3 | In this study |
| 259_UM_2018 | 2018 | Umbria | Sheep | LR735180 | B3 | In this study |
| 261_BA_2018 | 2018 | Basilicata | Sheep | LR735181 | B3 | In this study |
| 262_BA_2018 | 2018 | Basilicata | Sheep | LR735182 | B2 | In this study |
| 263_BA_2018 | 2018 | Basilicata | Sheep | LR735183 | B2 | In this study |
| 264_BA_2018 | 2018 | Basilicata | Sheep | LR735184 | B2 | In this study |
| 265_BA_2018 | 2018 | Basilicata | Sheep | LR735185 | B3 | In this study |
| 266_BA_2018 | 2018 | Basilicata | Sheep | LR735186 | B3 | In this study |
| 271_AB_2018 | 2018 | Abruzzo | Sheep | LR735187 | B3 | In this study |

| | | | | | | |
|---|---|---|---|---|---|---|
| 272_UM_2018 | 2018 | Umbria | Goat | LR735188 | B1 | In this study |
| 281_TO_2018 | 2018 | Toscana | Sheep | LR735191 | B3 | In this study |
| 276_PI_2018 | 2018 | Piemonte | Goat | LR735189 | B1 | In this study |
| 277_PI_2018 | 2018 | Piemonte | Goat | LR735190 | B1 | In this study |
| 282_TO_2018 | 2018 | Toscana | Sheep | LR735192 | B3 | In this study |
| 283_CA_2018 | 2018 | Calabria | Sheep | LR735193 | B2 | In this study |
| 284_UM_2018 | 2018 | Umbria | Goat | LR735194 | B1 | In this study |
| 286_UM_2018 | 2018 | Umbria | Sheep | LR735202 | A11 | In this study |
| 287_PI_2018 | 2018 | Piemonte | Goat | LR735203 | A8 | In this study |
| 288_MA_2018 | 2018 | Marche | Goat | LR735204 | B1 | In this study |
| 289_BA_2018 | 2018 | Basilicata | Goat | LR735205 | B1 | In this study |
| 290_BA_2018 | 2018 | Basilicata | Sheep | LR735206 | B3 | In this study |
| 296_UM_2018 | 2018 | Umbria | Sheep | LR735207 | B2 | In this study |
| 297_LZ_2018 | 2018 | Lazio | Sheep | LR735208 | A3 | In this study |
| 299_UM_2018 | 2018 | Umbria | Goat | LR735209 | B1 | In this study |
| 300_UM_2018 | 2018 | Umbria | Goat | LR735210 | A9 | In this study |
| 302_TO_2018 | 2018 | Toscana | Goat | LR735211 | B1 | In this study |
| 303_PI_2018 | 2018 | Piemonte | Goat | LR735212 | B1 | In this study |
| 304_UM_2018 | 2018 | Umbria | Goat | LR735213 | B1 | In this study |
| 307_LZ_2018 | 2018 | Lazio | Sheep | LR735214 | A24 | In this study |
| 309_PI_2018 | 2018 | Piemonte | Goat | LR735215 | A8 | In this study |
| 310_CA_2018 | 2018 | Calabria | Sheep | LR735216 | B2 | In this study |
| 311_CA_2018 | 2018 | Calabria | Sheep | LR735217 | B2 | In this study |
| 312_CA_2018 | 2018 | Calabria | Sheep | LR735218 | B2 | In this study |
| 317_UM_2019 | 2019 | Umbria | Sheep | LR735229 | B3 | In this study |
| 321_PU_2019 | 2019 | Puglia | Sheep | LR735230 | B2 | In this study |
| 322_UM_2019 | 2019 | Umbria | Sheep | LR735231 | B3 | In this study |
| 323_UM_2019 | 2019 | Umbria | Sheep | LR735232 | B3 | In this study |
| 324_UM_2019 | 2019 | Umbria | Sheep | LR735233 | A11 | In this study |
| 326_BA_2019 | 2019 | Basilicata | Goat | LR735234 | B1 | In this study |

| | | | | | | |
|---|---|---|---|---|---|---|
| 327_BA_2019 | 2019 | Basilicata | Goat | LR735235 | B1 | In this study |
| 328_UM_2019 | 2019 | Umbria | Sheep | LR735236 | B3 | In this study |
| 329_CM_2019 | 2019 | Campania | Goat | LR735237 | A20 | In this study |
| 332_BA_2019 | 2019 | Basilicata | Sheep | LR735238 | A9 | In this study |
| 333_BA_2019 | 2019 | Basilicata | Sheep | LR735239 | A9 | In this study |
| 334_BA_2019 | 2019 | Basilicata | Sheep | LR735240 | A9 | In this study |
